# Association between the blood concentrations of ammonia and carnitine/amino acid of schizophrenic patients treated with valproic acid

**DOI:** 10.1186/s13030-017-0101-0

**Published:** 2017-07-05

**Authors:** Masazumi Ando, Hideaki Amayasu, Takahiro Itai, Hisahiro Yoshida

**Affiliations:** 1 Department of Drug Metabolism and Disposition, Meiji Pharmaceutical University, 2-522-1 Noshio, Kiyose, Tokyo, 204-8588 Japan; 2 Department of Pharmacy, Heartful Kawasaki Hospital, 2-1-3 Shimonoge, Takatsu-ku, Kawasaki, Kanagawa 213-0006 Japan; 3 Division of Psychiatry, Heartful Kawasaki Hospital, 2-1-3 Shimonoge, Takatsu-ku, Kawasaki, Kanagawa 213-0006 Japan; 4 Department of Pharmacy, the 2nd Totsuka Kyoritsu Hospital, 579-1 Yoshida-cho, Totsuka-ku, Yokohama, Kanagawa 244-0817 Japan

**Keywords:** Hyperammonemia, Valproic acid, Carnitine, Glutamate, Glycine, CPS1, Mood stabilizer, Antipsychotics

## Abstract

**Background:**

Administration of valproic acid (VPA) is complicated with approximately 0.9% of patients developing hyperammonemia, but the pathogenesis of this adverse effect remains to be clarified. The aim of the present study was to search for mechanisms associated with VPA-induced hyperammonemia in the light of changes in serum amino acids concentrations associated with the urea cycle of schizophrenic patients.

**Method:**

Blood samples (10 mL) were obtained from 37 schizophrenic patients receiving VPA for the prevention of violent behaviors in the morning after overnight fast. Blood concentrations of ammonia, VPA, free carnitine, acyl-carnitine, and 40 amino acids including glutamate and citrulline were measured for each patient. Univariate and multivariate regression analyses were performed to identify amino acids or concomitantly administered drugs that were associated with variability in the blood concentrations of ammonia.

**Result:**

The blood ammonia level was positively correlated with the serum glutamate concentration (*r* = 0.44, *p* < 0.01) but negatively correlated with glutamine (*r* = −0.41, *p* = 0.01), citrulline (*r* = −0.42, *p* = 0.01), and glycine concentrations (*r* = −0.54, *p* < 0.01). It was also revealed that the concomitant administration of the mood stabilizers (*p* = 0.04) risperidone (*p* = 0.03) and blonanserin (*p* < 0.01) was positively associated with the elevation of the blood ammonia level.

**Conclusion:**

We hypothisized that VPA would elevate the blood ammonia level of schizophrenic patients. The observed changes in serum amino acids are compatible with urea cycle dysfunction, possibly due to reduced carbamoyl-phosphate synthase 1 (CPS1) activity. We conclude that VPA should be prudently prescribed to schizophrenic patients, particularly those receiving mood stabilizers or certain antipsychotics.

## Background

Valproic acid (VPA) is widely used for the treatment of generalized or partial epilepsy (e.g., absent seizures, tonic-clonic seizures) [1]. Recently, VPA has also been prescribed to patients with various psychological diseases, because it has been shown to be effective for ameliorating or preventing violent behaviors by patients with bipolar disorder, manic psychosis, and sometimes patients with schizophrenia [1]. It has been reported that one-third or more of schizophrenia patients admitted to hospitals are given VPA or other mood stabilizers in Japan [2] and the USA [3].

**Fig. 1:**
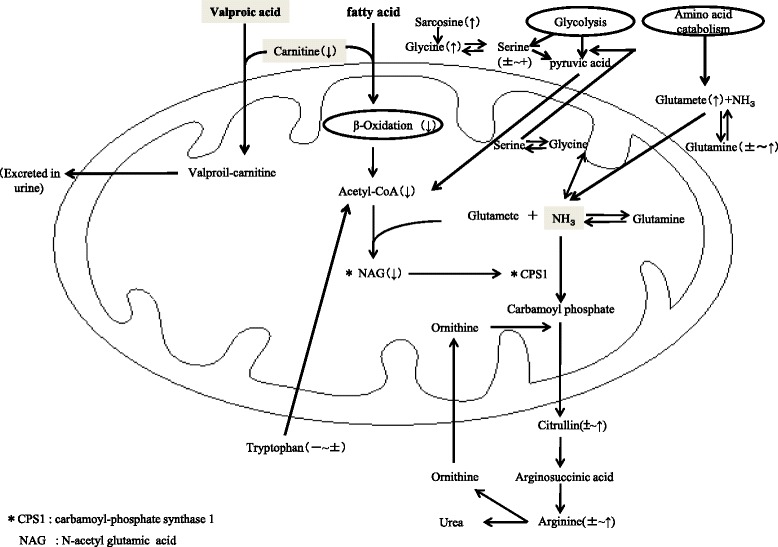
Schematic diagram illustrating the relation between the metabolism of valproic acid and those of amino acids associated with the urea cycle. The symbols 〔 ↑, ↓〕, − and ± indicate increase, decrease, stable, and either increase or decrease in the concentrations of the respective amino acids or substances. Abbreviations: CPS1: carbamoyl-phosphate synthase 1, NAG: N-acetyl glutamic acid

Hyperammonemia is a rare but severe adverse reaction associated with the administration of VPA. Clinical symptoms of VPA-induced hyperammonemia can range from mild disturbance of consciousness to coma [4–6]. At present, the etiology or pathogenesis of VPA-induced hyperammonemia is understood only partially. Briefly, VPA, a branched-chain carboxylic acid, is metabolized mainly by glucuronide conjugation in the cytosol, but it also undergoes β-oxidation in the mitochondria. VPA and other branched-chain fatty acids are transported into mitochondria via a common transporter of which activity is facilitated by conjugation with carnitine (Fig. 1) [7, 8]. Thus, when patients receive VPA the transport of carnitine-conjugated VPA into mitochondria may interfere with the transport of other fatty acids and thereby attenuate mitochondrial production of acetyl-CoA by the β-oxidation of fatty acids. Acetyl-CoA and glutamate are metabolized to N-acetyl glutamic acid (NAG) by N-acetyl glutamic acid synthase (NAGS) [9]. NAG is known to augment the activity of carbamoyl-phosphate synthase 1 (CPS1) by an allosteric mechanism [10]. Because ammonia is metabolized to carbamoyl phosphate by CPS1 and then enters the urea cycle, the VPA-induced attenuation of CPS1 activity may have a detrimental effect on ammonia metabolism.

Previous studies have reported that the administration of therapeutic doses of VPA to patients with epilepsy or bipolar disorder can cause hyperammonemia and alter serum carnitine and amino acid concentrations [11–14], whereas no attempts have been made to determine if VPA causes hyperammonemia when administered to patients with schizophrenia. Here, we report the relationships between blood ammonia concentration and the concentrations of free carnitine, acyl-carnitine, and amino acids, including glutamate and citrulline, in a cohort of schizophrenic patients receiving VPA. We hope to shed light on the mechanism of VPA-induced hyperammoninemia.

## Methods

### Participants

Schizophrenic patients who were admitted to Heartful Kawasaki Hospital between January 2013 and February 2014 participated in the present study after voluntarily giving written consent. The study protocol was approved by the Ethics Review Board of Meiji Pharmaceutical University (Approval No.: 2403). To protect patient privacy, personal information was carefully handled.

### Blood collection and measurements

Venous blood (10 mL) was collected in the morning after an overnight fast, before the administration of VPA. A portion of the sample was transferred to the clinical laboratory of our hospital for routine biochemical tests, and the remaining was divided into two samples. One was transferred to BML Co., Ltd. for determination of the blood ammonia concentration and the serum concentrations of free carnitine, acyl-carnitine, and 40 amino acids, including glutamate and citrulline. The other was transferred to the Pharmacokinetics Laboratory, Meiji Pharmaceutical University for determination of the serum VPA concentration. Briefly, blood samples were centrifuged at 3000 rpm for five minutes to isolate serum, which was then frozen and stored until measurement. For serum VPA analysis, a 5-fold volume of methanol was added to serum isolated from patients, mixed, rested for 30 to 40 min, and centrifuged at 12,000 rpm for 1 min for deproteinization. VPA was assayed with high-performance liquid chromatography (HPLC) with mass spectrometry (LC-MS) detection using the LC-MS 2020 system (Shimadzu, JAPAN). Assay conditions were as follows: column, C180DS (2 × 50 mm); mobile phase, a solution consisting of 20 mM ammonium formate and acetonitrile; flow velocity, 0.2 mL/min; mode of analysis, SIM measurement; and range of analysis, m/z 143.3.

### Statistical analysis

Because we did not measure the amino acid concentrations of healthy subjects, we adopted the reference ranges of the respective amino acids that were established for healthy subjects by the laboratory of BML Co. Ltd. Multivariate regression analysis was conducted to determine if any of the serum amino acids and other substrates are significant covariates of blood ammonia concentration. Analysis was also conducted to determine if the administration of mood stabilizers or antipsychotics as a group would be associated with the variability of blood ammonia concentration. We also conducted a multivariate regression analysis to examine whether or not any of the individual antipsychotics would exert an independent influence on the variability of the blood concentration of ammonia. Multivariate regression analysis was performed using JMP® software (ver. 11). A *p*-value of less than 0.05 was considered statistically significant.

## Results

Thirty-seven schizophrenic patients (24 male and 13 female aged from 37 to 77 years) who received VPA participated in the present study. Their body weights ranged from 34 to 82 kg, and the mean (± SD) dose of VPA was 12 ± 5 mg/kg, equivalent to 1481 ± 1347 mg/day of chlorpromazine [15]. All patients consumed a regular hospital diet consisting of adequate amounts of calorie, protein and vitamins. Table 1 shows the blood concentrations of ammonia, aspartate transaminase (AST), alanine transaminase (ALT), and VPA. Elevation of the laboratory data exceeding 30% or greater than the upper limits of the respective reference ranges was considered clinically significant. While 30% of the patients showed clinically significant elevation of blood ammonia, less than 11% of them showed clinically meaningful elevation of AST or ALT. Ninety-five percent of the patients had serum VPA concentration within the therapeutic range.

**Table 1:** Mean (±SD) values for blood ammonia concentration, VPA doses, and liver function as well as percentage of patients exhibiting clinically significant elevations of the respective laboratory data

constituent	Unit	Total (*n* = 37)			
		Reference range	Mean ± SD of patients	Below Reference range %	Patients exhibiting values of 1.3-times or greater than the upper limits of the reference range (%)
ammonia	μg/dL	18-70	64 ± 19	0	30
AST	IU/L	7-38	21 ± 7	0	5
ALT	IU/L	4-44	23 ± 15	0	11
VPA concentration	μg/mL	50-100	51 ± 24	54	5

Abbreviations:* AST* Aspartate transaminase, *ALT* Alanine transaminase, *VPA* valproic acid

Table 2 shows the serum concentration of amino acids and related constituents that were measured in the present study. Using the reference values, we assessed whether or not the serum amino acid profiles of patients receiving VPA would be altered as compared to those of healthy subjects. A considerable percentage of patients showed serum concentrations of several amino acids that were 1.3-times greater than the upper limit of the reference ranges: glutamate (68%), glutamine (38%), glycine (38%), α-amino-n-butyric acid (70%), cystathionine (73%), and taurine (35%). In contrast, the serum concentrations of free carnitine, acyl-carnitine, and tryptophan were reduced as compared to the respective reference ranges. Univariate regression analysis revealed that the blood concentration of ammonia was correlated positively with that of glutamate (*r* = 0.44, *p* < 0.01) and tryptophan (*r* = 0.35, *p* = 0.03). Conversely, the blood concentration of ammonia was correlated negatively with that of glutamine (*r* = −0.41, *p* = 0.01), citrulline (*r* = −0.42, *p* = 0.01), and glycine (*r* = −0.54, *p* < 0.01).

**Table 2:** Serum concentrations of amino acids, free carnitine, acyl-carnitine obtained from patients and the respective reference ranges, as well as the correlations of their concentrations with blood ammonia concentrations

constituent	Unit	Total (*n* = 37)				correlation coefficient	
		Reference range	Mean ± SD	Patients exhibiting below the lower limits of the reference range (%)	Patients exhibiting values of 1.3-times or greater than the upper limits of the reference range (%)	*r* value	*p* value
Free Carnitine	μmol/L	36-74	33 ± 13	41	0	0.02	0.91
Acyl-Carnitine	μmol/L	6-23	8 ± 4	38	0	−0.24	0.15
1-Methyl-histidine	nmol/mL	<9.1	5 ± 5	0	8	−0.09	0.66
3-Methyl-histidine	nmol/ml	TRA-8.2	4 ± 2	0	5	−0.30	0.08
Alanine	nmol/mL	258.8-615.2	458 ± 130	3	8	0.24	0.15
Anserine	nmol/ml	ND	ND	0	0	-	-
Arginine	nmol/mL	31.8-149.5	128 ± 32	0	27	−0.27	0.10
Asparagine	nmol/ml	43.8-90.6	56 ± 11	19	0	−0.23	0.17
Aspartic acid	nmol/mL	TRA-7.2	5 ± 2	0	5	0.08	0.63
Carnosine	nmol/mL	ND	ND	0	0	-	-
Citrulline	nmol/mL	17.9-48.0	45 ± 12	0	27	−0.42	0.01*
Cystathionine	nmol/mL	ND	2 ± 0	0	73	−0.09	0.66
Cystin	nmol/mL	4.7-34.8	19 ± 11	3	14	−0.25	0.14
Ethanolamine	nmol/mL	TRA-10.5	7 ± 2	0	3	0.15	0.37
Glutamate	nmol/mL	12.2-82.7	122 ± 59	0	68	0.44	〈0.01*
Glutamine	nmol/mL	418.0-739.8	700 ± 128	0	38	−0.41	0.01
Glycine	nmol/mL	140.4-427.3	394 ± 102	0	38	−0.54	〈0.01*
Hdroxylysine	nmol/mL	ND	ND	0	ND	-	-
Histidine	nmol/mL	63.0-105.2	94 ± 15	0	27	0.00	0.99
Homocystine	nmol/mL	ND	ND	0	0	-	-
Hydroxyproline	nmol/mL	TRA-18.8	17 ± 5	0	27	−0.30	0.10
Isoleucine	nmol/mL	37.0-100.4	78 ± 15	0	8	−0.07	0.67
Leucine	nmol/mL	74.2-169.1	134 ± 25	0	8	0.05	0.76
Lysine	nmol/mL	125.7-281.9	237 ± 44	0	22	−0.30	0.08
Methionine	nmol/mL	15.5-38.6	30 ± 5	0	8	−0.14	0.40
Ornithine	nmol/mL	42.6-141.2	86 ± 25	0	3	−0.05	0.78
Phenylalanine	nmol/mL	43.5-79.8	59 ± 9	0	3	0.02	0.91
Phosphoethanol-amine	nmol/mL	TRA	TRA	0	0	-	-
Proline	nmol/mL	71.3-373.0	289 ± 241	0	11	−0.16	0.36
Sarcosine	nmol/mL	TRA	8 ± 3	0	51	−0.34	0.15
Serine	nmol/mL	91.5-186.4	168 ± 34	0	24	−0.04	0.81
Taurine	nmol/mL	46.4-128.2	110 ± 43	3	35	−0.15	0.37
Threonine	nmol/mL	74.2-216.1	144 ± 35	0	3	−0.15	0.39
Tryptophan	nmol/mL	36.2-79.3	37 ± 10	59	0	0.35	0.03*
Tyrosine	nmol/mL	38.4-89.4	72 ± 18	0	16	0.18	0.28
Valine	nmol/mL	156.2-360.4	248 ± 41	0	0	−0.02	0.89
α-aminoadipic acid	nmol/mL	ND	ND	0	0	-	-
α-amino-n-butyric acid	nmol/mL	8.1-31.0	35 ± 8	0	70	−0.10	0.06
β-Alanine	nmol/mL	TRA-11.8	2 ± 1	0	0	0.05	0.88
β-Amino-iso-butyric acid	nmol/mL	<5.9	3 ± 2	0	5	−0.18	0.32
γ-Amino-nbutyric acid	nmol/mL	ND	ND	0	0	-	-

The correlation between the concentration of ammonia and each item is presented. A *p*-value of 0.05 was regarded as significant

*TRA* Trace

*ND* Not detectable

*: *p* < 0.05

Table 3 shows the results of multivariate regression analysis conducted to identify significant covariates for blood ammonia concentration. The results indicated that the blood concentration of ammonia was positively correlated with that of glutamate (*p* < 0.01), valine (*p* < 0.01), and cysteine (*p* = 0.04) but negatively correlated with that of α-amino-n-butyric acid (*p* < 0.01), ethanolamine (*p* < 0.01), and 3-methylhistidine (*p* < 0.01).

**Table 3:** Results of multivariate regression analysis performed for identifying significant covariates of blood ammonia concentrations

constituent	Unit	Total (*n* = 37)		
		Estimate	*F* value	*p* value
Dose of VPA	mg/day	0	0.53	0.49
VPA concentration	μg/mL	0	1.13	0.32
Free Carnitine	μmol/L	0	0.14	0.72
Acyl-Carnitine	μmol/L	0	0.07	0.80
Cysteine	nmol/mL	0.26	6.01	0.04*
Ethanolamine	nmol/mL	−5.45	21.03	<0.01*
Glutamate	nmol/mL	0.15	34.07	<0.01*
Valine	nmol/mL	0.09	14.46	<0.01*
α-amino-n-butyric acid	nmol/mL	−0.49	19.79	<0.01*
3-metyl-Histidine	nmol/mL	−1.57	20.63	<0.01*

VPA: valproic acid

*: *p* < 0.05

Table 4 shows the results of the multivariate regression analysis conducted to examine whether or not the administration of mood stabilizers or antipsychotics with VPA would be associated with variability of blood ammonia concentration. All patients were given antipsychotic drugs and mood stabilizers. When we examined whether or not the administration of mood stabilizers or antipsychotics as a group had an influence on blood ammonia concentration, the administration of mood stabilizers (*p* = 0.04), but not antipsychotics, augmented blood ammonia concentration. However, when we evaluated the independent contribution of each drug with a multivariate regression analysis, we found that only risperidone (*p* = 0.03) and blonanserin (*p* < 0.01) were significantly associated with elevation of the blood concentration of ammonia (Table 5).

**Table 4:** Influence of the number of antipsychotics or mood stabilizers on the concentration of ammonia (regression analysis)

	Total (*n* = 37)			
	N	Estimate	*F* value	*p* value
Antipsychotic drug	37	0	0.62	0.44
Mood stabilizer	37	12.94	4.66	0.04*

*: *p* < 0.05

**Table 5:** The results of multivariate regression analysis exhibiting the influence of individual antipsychotics on blood ammonia concentrations

	Total (*n* = 37)			
	N	Estimate	*F* value	*p* value
zotepine	22	0	1.06	0.31
haloperidol	18	0	0.21	0.65
chlorpromazine	18	0	0.66	0.42
risperidone	13	−6.70	5.17	0.03*
fluphenazine	10	0	0.11	0.75
levomepromazine	6	0	0.77	0.39
sultopride	5	−7.46	3.35	0.77
olanzapine	5	0	0.05	0.83
quetiapine	5	0	0.14	0.71
nemopride	4	0	0.05	0.82
aripiprazole	4	0	1.15	0.29
bromperidol	3	0	0.05	0.83
blonanserin	3	−15.14	7.80	<0.01*
propericyazine	2	−12.62	3.61	0.67
perospirone	2	0	0.40	0.53
pimozide	1	0	0.85	0.37
paliperidone	1	0	<0.01	0.97

*: *p* < 0.05

Scrutinizing the concomitant medications of the 37 schizophrenic patients who participated in the study, it was found that none had received corticosteroids or amino acids or were suffering from malnutrition.

## Discussion

In the present study, we revealed that about one-third of the schizophrenic patients receiving VPA showed an elevated blood ammonia concentration: 30% of the patients showed a blood ammonia level of 1.3 times or higher than the upper limit of the reference values (Table 1). In addition, 68%, 38%, and 38% of the participants showed a clinically significant elevations of the serum concentration of glutamate, glutamine, and glycine, respectively (Table 2). In addition, there was a positive correlation between the blood concentrations of glutamate and ammonia (Table 3). Conversely, there were negative correlations between the blood ammonia concentration and that of glutamine and glycine (Table 3). In addition, 41% of the patients showed a reduced serum free carnitine concentration below the lower limit of the reference range. These findings appear largely compatible with the hypothesis that reduced serum carnitine concentration can be attributed to the augmented consumption of carnitine by conjugation with VPA and transport into mitochondria. If the cytosolic carnitine pool available for the transport of other branched chain fatty acids is depleted by VPA, the β-oxidation of these fatty acids and resultant production of acetyl-CoA might have been impaired. Consequently, the production of NAG from acetyl-CoA and glutamate might also have been impaired [7, 8]. The significant elevation of the serum glutamate level might have been due to an attenuated production of NAG from acetyl-CoA and glutamate. Because NAG stimulates the activity of carbamoyl-phosphate synthase 1 (CPS1), which is a key enzyme of the ATP-dependent synthesis of carbamoyl phosphate from ammonia or glutamine and bicarbonate [10], the shortage of NAG would impair the subsequent reactions in the urea cycle.

The idea that VPA inhibits the activity of carbamoyl-phosphate synthase I (CPS1) was proposed for the first time in literature by Vazquez and others [16]. They studied the VPA-induced hyperammonemia of 11 pediatric patients with epilepsy only in the light of altered plasma concentrations of carnitine and acetylcarnitine. In the present study, however, we studied the alerted ammonia metabolism of patients receiving VPA not only in the light of changes in the carnitine/acetylcarnitine concentration but also of other amino acids that might also be involved in altered ammonia metabolism. In this context, the present study has expanded and deepened the original concept of the inhibitory effects of VPA on the activity of CPS1 and hyperammonemia.

We performed multivariate regression analysis to investigate the influence of the dose and plasma concentrations of VPA, free carnitine, acyl-carnitine, and six other amino acids on the blood concentration of ammonia (Table 3). No significant correlations were observed between the blood ammonia level and the VPA dose or, serum concentration of VPA, free carnitine, and acyl-carnitine. This finding contradicts that of a previous study [17], but was consistent with others [18, 19]. At present, reports on the relation between the plasma concentrations of VPA and ammonia are conflicting, In contrast, the serum concentrations of cysteine, ethanolamine, glutamate, valine, glutamate, α-amino-n-butyric acid, and 3-methyl-histidine were significantly correlated with blood ammonia concentration. Of them, glutamate had the greatest contribution among other factors to the variability of blood ammonia concentration. Alterations in serum cysteine and valine concentration may have been influenced by elevated glutamate concentration, because glutamate is synthesized by tansamination of these amino acids by glutamate-α-ketoglutaric acid at the time of catabolism (Fig. 1) [20].

While the genetic polymorphism of CPS1 is associated with reduced enzyme activity [21, 22], concomitantly administrated medications, such as antiepileptic drugs with enzyme inducing property (e.g., carbamazepine, phenobarbital, phenytoin), were shown to alter CPS1 activity [10]. Previous studies have suggested that the concomitant use of VPA with an antipsychotic, risperidone, may be a risk factor for hyperammonemia through competition for drug protein binding [11–13]. However, it remains to be clarified whether or not the combination of certain antipsychotics reduces CPS1 activity. Since the schizophrenia patients investigated in this study received both antipsychotics and mood stabilizers, we performed a multivariate analysis to study whether or not the co-administration of these drugs would have affected the blood ammonia concentration. The results indicated that the concomitant administration of mood stabilizers is associated with increased blood ammonia concentration, but that the administration of antipsychotics as a whole is not (Table 4). VPA had been administered to all patients, some of whom were receiving lithium (*n* = 5) or carbamazepine (*n* = 5). Because the 17 different antipsychotics prescribed are quite different in physicochemical and pharmacological properties, we undertook a multivariate analysis to determine if any of them independently influenced the blood ammonia level (Table 5). We found that concomitant administration of risperidone or blonanserin was associated with a reduced blood concentration of ammonia, which somewhat conflicts with the results reported by previous studies [11–13], our data will need to be confirmed in a study with a larger number of patients: the number of patients receiving blonanserin with VPA was small (*n* = 3) in the present study.

There is a possibility that other drugs administered for the treatment of non-psychiatric diseases (e.g., corticosteroids) or the poor nutritional status of the patients might influence the blood ammonia concentration [23–26]. However, none of our patients received corticosteroids. In addition, they orally consumed a standard hospital diet and received no parenteral amino acid supplementation. Because ammonia is metabolized by the urea cycle in the liver, liver dysfunction might have been associated with an increase in the blood concentration of ammonia. However, none of the patients had severe liver dysfunction (Table 1), therefor it would be only a remote possibility that liver dysfunction was associated with our findings. We found that the serum levels of glutamate and glycine were elevated in this cohort of schizophrenic patients receiving VPA. Because glutamate is an agonist of N-methyl-D-aspartate (NMDA) receptors in the cerebral excitatory nerves and glycine is a co-agonist of NMDA receptors [27], the administration of VPA might have offset the therapeutic effects necessary for the prevention of violent behaviors by these patients.

Currently, VPA is frequently used for ameliorating and/or preventing the violent behaviors of patients with schizophrenia [1–3]. The effects of VPA on blood ammonia concentration observed for schizophrenic patients are largely compatible with those reported for patients with other psychiatric disorders and for those with epilepsy [10–14]. However, caution should be exercised in drawing conclusions, because there are substantial differences in the VPA dose, concomitant medications, and the genetic background of the two groups of patients. For patients with chronic schizophrenia receiving VPA, dose-reduction or discontinuation is not often discussed, whether or not violent behaviors have been observed. As a result, the patients often receive polypharmacy. There is no evidence that polypharmacy is more effective than monotherapy [28]. The present study revealed that VPA may alter the metabolism of fatty acid and the activity of the urea cycle in such a way that the blood ammonia level is increased in 30% of the schizophrenic patients. Based upon our data, we feel that it would be prudent to reduce the VPA dose or to discontinue it, if possible. For patients with a previous history of epilepsy, the administration of levocarnitine may be worth trying, because it may activate β-oxidation and the activity of CPS1, thereby activating the urea cycle and reducing the blood concentration of ammonia [29, 30]. We recommend the monitoring of the blood ammonia level of schizophrenic patients receiving VPA.

## Conclusions

In the present study we found that the administration of VPA increased the blood concentrations of ammonia, glutamate, and glycine in some schizophrenic patients receiving mood stabilizers and/or antipsychotics. VPA should be administered carefully to patients with schizophrenia, because it is frequently selected to prevent the appearance of violent behavior. We consider it prudent to refrain from too easily administering VPA to schizophrenic patients for the prevention of violent behaviors.

## Abbreviations

VPA: Valproic acid
NAG: N-acetyl glutamic acid
NAGS: N-acetyl glutamic acid synthase
CPS1: carbamoyl-phosphate synthase 1
NMDA: N-methyl-D-aspartate
